# Early Implant Failure: A Meta-Analysis of 7 Years of Experience

**DOI:** 10.3390/jcm13071887

**Published:** 2024-03-25

**Authors:** Radu Ionut Grigoras, Adina Cosarca, Alina Ormenișan

**Affiliations:** 1 IOSUD Doctoral School, George Emil Palade University of Medicine, Pharmacy, Science, and Technology of Targu Mures, 540142 Targu Mures, Romania; radu.grigoras@umfst.ro; 2 Department of Oral and Maxillo-Facial, George Emil Palade University of Medicine, Pharmacy, Science, and Technology of Targu Mures, 540142 Targu Mures, Romania; alina.ormenisan@umfst.ro

**Keywords:** dental implant, osseointegration, peri-implantitis

## Abstract

**Background:** The use of dental implant rehabilitation in the treatment of complete and partial edentulism has become an integral treatment today. This treatment is performed on healthy patients, but in some situations, also on those with associated general ailments. The presence of associated conditions increases the degree of difficulty of this type of treatment and tests the doctor’s ability to manage the clinical case. The purpose of the study was to perform a meta-analysis of dental implants inserted over seven years and evaluate early implant failure in correspondence with age, sex, region of insertion, type of implant, and general state of health. **Methods:** A retrospective study was performed over 7 years of experience. For the study, 213 patients who fit the established inclusion criteria were selected. Patients were grouped taking into account age, sex, the type of implant used, and general associated conditions. The collected data were analyzed using IBM SPSS STATISTICS 25.0 for windows **Results:** There were no highlighted situations in which the rejection of the dental implant occurred 10 days postoperatively or later during the healing period. **Conclusions:** Our results confirm and strengthen the existing data in the specialized literature, especially those related to the loss of implants in patients with associated general diseases.

## 1. Introduction

Dental implantation therapy is a viable solution for replacing teeth that can no longer be restored by dental, endodontic, or prosthetic methods with a high degree of success in oral rehabilitation. The field of implantology has presented, in recent years, considerable development, which has led to an increase in the number of inserted dental implants and the improvement in the procedures for inserting dental implants [1].

Implant therapy is an effective method of treatment for partially or totally edentulous patients, but there is no total success in this treatment. Failures of implant therapy can be divided into two large categories: early loss of the dental implant and late loss of the dental implant. The early loss of a dental implant occurs when strong intimate contact between the bone and the working implant is not obtained, which can be determined by certain local factors (the design of the dental implant, the degree of aggressiveness of the implant coils, the quantity and quality of the alveolar bone, history of periodontal or systemic diseases, increased cholesterol levels associated with reduced vitamin D values, or certain general diseases triggered after the insertion of dental implants), while the late loss of a dental implant is attributed to the microbial factor, parafunctions, and prosthetic errors. Likewise, other external factors can negatively influence implant therapy: the operative technique, the surgeon’s experience, and the type of materials used for bone addition. The American Academy of Implant Dentistry defined the osseointegration of dental implants as “contact established without the interposition of nonbone tissue between normal remodeled bone and an implant, entailing a sustained transfer and distribution of load from the implant to and within the bone tissue” [2].

The successful replacement of a natural tooth with the help of tissue-integrated implants is solely based on successful osseointegration [3].

Osseointegration is a direct structural and functional connection between bone and the surface of a load-carrying implant [4]. A lack of primer stability, surgical trauma, and infection seem to be the most important causes of early implant failure. Despite the high success rates and stability of dental implants, failures do occur [5]. Implant failure has been categorized as early and late in retrospective studies according to different cutoff time points, such as at the time of abutment connection [6], at the time of loading [7], within several weeks after the placement of the final prosthesis [8], or at the time of the first year after loading [9]. Early implant failure is usually attributed to biological factors, surgical trauma, and impaired healing that result in a failure to achieve osseointegration [10]. 

Early implant failure means an implant showing clinical mobility before the placement of a final prosthesis. This is usually because of biological problems where the body does not accept the implant. It is called the “rejection” of the dental implant. Early implant failure may be linked to immunological, genetic, and immunological variables [11].

Most implant failures have been reported in the maxilla, with almost three times as many implant losses as in the mandible [12]. The success of dental implants results from various factors such as the skill and proficiency of the practician. It was suggested that the dentist’s years of experience, implant training, and postgraduate specialization might affect the knowledge, attitude, and method of practice of dental implants [13]. The integrity of the hard and soft tissues around dental implants is the key to dental implant longevity [14,15].

The purpose of this study was to perform a meta-analysis of dental implants inserted over 7 years and evaluate early implant failure in correspondence with age, sex, region of insertion, type of implant, and general state of health.

## 2. Materials and Methods

We conducted a observational study over a period of 7 years, between 2016 and 2023. The study was carried out in a private dental practice Detal Dor, Suceava Street 53, in Targu Mures, Romania.

The treatment possibilities as well as any associated problems that could have arisen from the implantation therapy were presented, and all the patients gave their signed informed consent for participation in this study. All then data were collected with Microsoft Ofiice Profesional Plus 2019, version 2402. The study was conducted in accordance with the Declaration of Helsinki and approved by the Institutional Review Board (or Ethics Committee) of George Emil Palade University of Medicine, Pharmacy, Science, and Technology of Târgu Mureș, Romania, number 972/18.06.2020.

The inclusion criteria used in this study were as follows:

Patients who required at least one dental implant restoration;

Patients aged between 21 and 78 years old;

Patients with a good general health status;

Patients with compensated morbidity such as diabetes, cardiovascular disease, or arterial hypertension.

The exclusion criteria for the study were as follows:

Patients with general conditions that were not medically compensated;

Patients with general conditions that contraindicated implant treatment such as cancers in the oral–maxillofacial area;

Patients who underwent chemotherapy or radio-therapy in the last year.

The patients included in this study benefited from general medical examination, dental examination, radiological examination that included OPG, and cone beam computer tomography examination. For all the patients, screw-type implants were used with conical connections and platform and non-platform switching.

### 2.1. Methodology of Implant Therapy

The implant treatment was performed in a dental practice specifically equipped for maxillofacial surgery, using the following medical protocol: the lavage of the oral cavity with Curasept™ ADS 5020 CU 0.20% mouthwash solution (CURAPROX-Shop Schweiz, Amlehnstrasse 22, Postfach 1063, CH-6011 KriensSwitzer land CuraProx, Kriens, Switzerland) for 45 s.

Local–regional anesthesia was achieved using Ubistesin™ forte 4%, Articaine™ with 1:100,000 adrenaline 3M Oral Care–3MEspe USA (3M Oral Care, 3MESPE, St. Paul, MN, USA) (no other methods of sedation and analgesia were used in general) in the surgical interventions that were performed.

**Figure 1:**
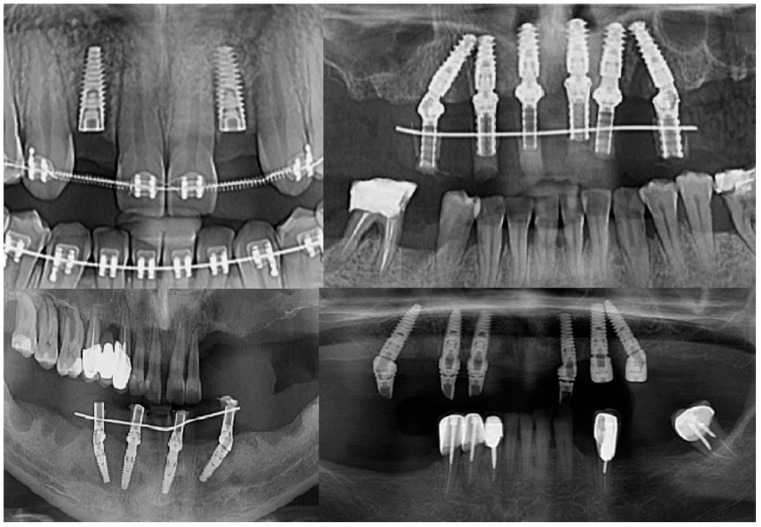
Radiological images presenting different clinical cases included in the study.

Intraoperatively, the dental implants were screwed into the alveolar bone according to the manufacturers’ indications, being applied over the dental implant with either a covering screw or healing abutment or multiunit abutment (Figure 1). The postoperative wound was sutured with Dafilon™ 4/0 suture thread (BBraun Hessen, Germany BBraun, Spangenberg, Germany) and Dafilon™ 5/0 suture thread (BBraun Hessen, Germany BBraun, Spangenberg, Germany).

**Figure 2:**
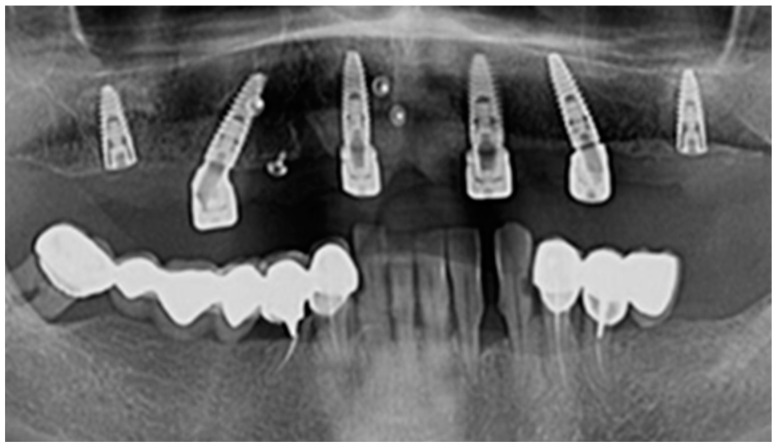
Radiological examination of a bone augmentation clinical case.

In the lateral areas of the jaws where the bone support was not enough, sinus lifting using BioOss (Geistlish Pharma AG, Baden-Baden Germany) and the resorbable membrane Bio Guide (Geistlish Pharma AG, Baden-Baden Germany) was used (Figure 2).

Post-surgery, patients benefited from antibiotic treatment: 1000 mg of Augmentin™ (GlaxoSmithGKline, Brentford, UK) twice a day, starting the day before implant treatment. In the case of allergy to penicillin, patients were administered 300 mg of Clindamycin (Pfizer Inc., New York, NY, USA) three times a day; an anti-inflammatory like 500 mg of paracetamol (Bayer AG, Leverkusen, Germany) twice a day for 7 days was also indicated. To maintain oral hygiene, patients were advised to carry out dental brushing with a dental brush with ultra-soft bristles, alongside the use of Parodontax 0.2% mouthwash (GlaxoSmithGKline, Brentford, UK) for 2 weeks.

**Figure 3:**
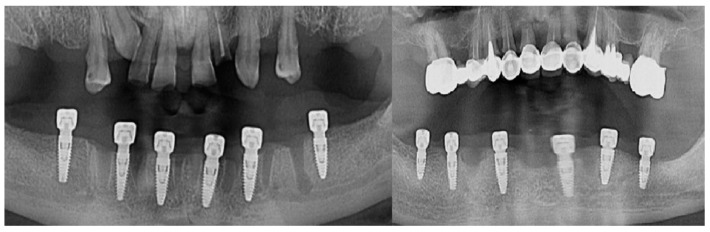
Radiological images of clinical cases during the follow-up period.

The follow-up of the patients was made at 1 month, 3 months, and 6 months from the moment the suture threads were removed. Retro-alveolar and OPG X-rays were performed in isometric and orthoradial incidences using the Belot method [16] (Figure 3). In cases where any kind of suppurated complications occurred less than a month after the insertion, the dental implant was removed.

Later in the study, the implants were examined for tissue integration according to the parameters defined by Buser [17], so the integration was considered successful if the following parameters were met:

The absence of recurrent peri-implantation infection with suppuration;

The absence of pain or foreign body sensation;

The absence of continuous radio-transparence around the implant;

The absence of any detectable mobility of the implant.

These criteria have proven to be effective in defining the success of an implant system and evaluating long-term results in clinical trials [17].

In this study, the failure of a dental implant was defined as infection of the hard and soft peri-implantation tissues; the appearance of a radio-transparent area around the dental implant; bone resorption accentuated more than 1 mm in the first year after loading it with prosthetic work; persistent pain; purulent secretion; and/or foreign body sensation.

### 2.2. Statistical Analysis

The collected data were analyzed using IBM SPSS STATISTICS 25.0 for Windows™.

## 3. Results

**Figure 4:**
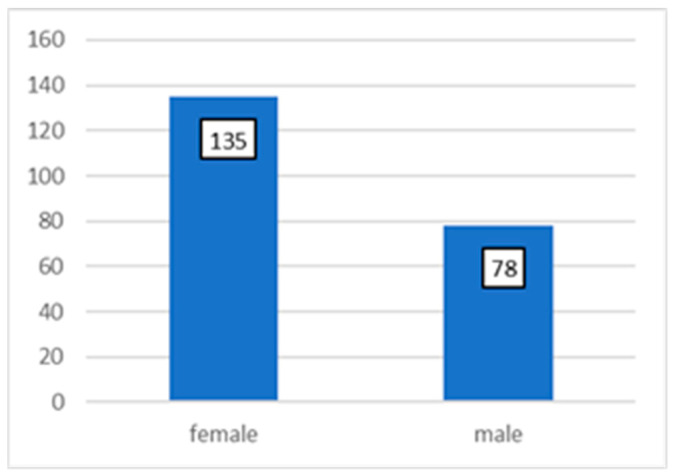
Distribution by gender.

A total of 213 patients were selected for the study (Figure 1), and dental implants were inserted in these participants; 78 patients were male and 135 patients were female (Figure 4).

A total of 810 dental implants were inserted over a period of 7 years (Table 1); 421 dental implants were inserted in women (52.0%) and 389 dental implants were inserted in men (48.0%) (Table 2).

From the total of 810 dental implants inserted, 194 dental implants were inserted in the right upper jaw (24.0%), 193 dental implants were inserted in the left upper jaw (23.8%), 236 dental implants were inserted in the right lower jaw (29.1%), and 187 dental implants were inserted in the left lower jaw (23.1%) (Table 3).

In men, 96 (11.8%) dental implants were inserted in the right upper jaw, and 78 (9.6%) dental implants were inserted in the left. In men, 116 (14.3%) dental implants were inserted in the right maxilla and 99 (12.2%) dental implants in the left side.

In women, 98 (12.1%) dental implants were inserted in the right upper jaw, and 115 (14.2%) dental implants were inserted in the left side. In women, 120 (14.8%) dental implants were inserted in the right lower jaw and 88 (10.9%) implants in the left side (Table 4).

From the total number of 810 dental implants inserted, in the upper jaw, 103 implants required vestibular bone addition (12.7%), and in the lower jaw, 63 dental implants (7.8%). A total of 365 dental implants were inserted without the need for vestibular bone addition in the upper jaw (45.1%), and this was the case for 279 in the lower jaw (34.4%) (Table 5).

In the case of men, 45 dental implants inserted in the upper jaw required vestibular bone addition (5.6%), while 136 dental implants did not require vestibular bone addition (16.79%).

In women, in the upper jaw, 58 implants required vestibular bone addition (7.16%), and 229 implants were inserted without the need for bone addition (28.27%).

A total of 23 dental implants inserted in the lower jaw required vestibular bone addition (2.8%), while 105 dental implants did not require vestibular bone addition (13.00%).

In women, in the lower jaw, 40 implants required vestibular bone addition (4.9%), and 174 implants were inserted without the need for bone addition (21.5%) (Table 6).

At the level of the upper jaw, in the case of men, 197 dental implants (24.3%) without addition in the maxillary sinus and 81 dental implants (10.%) with addition in the maxillary sinus were inserted in the lateral areas. In the case of women, 426 dental implants without bone addition (52.6%) and 106 dental implants with bone addition were inserted in the maxillary sinus (13.1%) (Table 7).

From the total number of inserted implants to which bone addition was made, 39 (4.8%) implants were inserted in the right maxillary sinus with bone addition in men, and 59 (7.3%) implants were inserted in women on the same side. In the left maxillary sinus with bone addition, 42 (5.2%) dental implants were inserted in men and 47 (5.8%) implants in women (Table 8).

In certain situations, after the insertion of the dental implants, it was possible to load them with provisional prosthetic works. A total of 9 (15.5%) dental implants were inserted in the upper jaw in men and 12 (20.7%) in the mandible, and in women, 12 (20.7%) dental implants were inserted in the upper jaw and 25 in the mandible (43.10%) (Table 9). There was no implant that was lost early after being loaded with provisional work.

From the total of 810 inserted dental implants, 22 dental implants did not osseointegrate (2.71%). In men, three dental implants did not show osseointegration in the upper jaw (13.6%), and five did not in the mandible (22.7%). In women, eight dental implants were not osseointegrated in the maxilla (36.4%), and six were not in the mandible (27.3%) (Table 10).

In men, in the upper jaw, three (13.6%) implants were not osseointegrated and external sinus lifting was performed, one (4.5%) implant was implanted with vestibular bone addition, and one (4.5%) implant did not require bone addition. In men, in the lower jaw, three (13.6%) implants did not osseointegrate, to which vestibular bone addition was made, and there were two (9.1%) implants to which no bone addition was needed. In women, in the upper jaw, four (18.2%) implants were not osseointegrated and external sinus lifting was performed, two (9.1%) implants were fitted with vestibular bone addition, and one (4.5%) implant did not require bone addition. In women, in the lower jaw, four (18.2%) implants did not osseointegrate, to which vestibular bone addition was made, and there was one (4.5%) implant to which no bone addition was needed. (Table 11).

The type of dental implants that were used for the study were platform switching and non-platform switching. In men, 87 platform-switching implants (10.7%) and 89 non-platform-switching implants (11.0%) were used in the upper jaw, and in women, 99 platform-switching implants (12.2%) and 93 non-platform-switching implants (11.5%) were used. In men, 95 platform-switching implants (11.7%) and 109 non-platform-switching implants (13.5%) were used in the lower jaw, and in women, 109 implants with switching platforms (13.5%) were inserted, and 129 non-platform switching implants (15.9%) were inserted (Table 12).

With regard to the number of non-osseointegrated implants, 17 (77.3%) implants were in patients with affected general states of health, and 5 (22.7%) implants were inserted in patients without health issues.

Two non-osseointegrated implants in men with diabetes (9.1%), one implant in a man with cardiovascular disease (4.5%), and one non-osseointegrated implant in a man with arterial hypertension (4.5%) were observed in the upper jaw. In the case of women, only one (4.5%) non-osseointegrated implant was observed both for cardiovascular disease and in the case of arterial hypertension.

In the case of the mandible, we observed three (13.6%) non-osseointegrated implants in men with diabetes and one (4.5%) non-osseointegrated implant in the case of arterial hypertension, and we observed five (22.7%) non-osseointegrated implants in women with diabetes and two (9.1%) non-osseous implants in women with arterial hypertension (Table 13).

The dental implants that were used in the study had different diameters (a narrow platform <3.5 mm, a standard platform between 3.6 and 4.5 mm, and a wide platform >4.5 mm) and different lengths (8 mm, 10 mm, 11.5 mm, 13 mm, and 16 mm).

From the number of non-osseointegrated dental implants, in the case of men in the upper jaw, it was found that three implants with a narrow platform (13.6%) and two implants with a standard platform (9.1%) were not osseointegrated. In the case of women in the upper jaw, two (9.1%) implants with a narrow platform and two (9.1%) with a standard platform did not osseointegrate.

In the lower jaw, in the case of men, three (13.6%) implants with a narrow platform and four (18.2%) with a standard platform did not osteintegrate, and in women, four (18.2%) implants with a narrow platform and two (9.1%) with the standard platform did not osseointegrate (Table 14).

From the total number of implants that did not osseointegrate in relation to the length of the implant, nine dental implants (40.9%) were 10 mm long, six dental implants (27.3%) were 8 mm long, four dental implants (18.2%) had lengths of 11.5 mm, two dental implants (9.1%) had lengths of 13 mm, and one dental implant (4.5%) had a length of 16 mm (Table 15).

In the studied group, we compared the diameters of non-osseointegrated implants between gender by applying the T Sample test with a 95% confidence interval, and we obtained a statistically significant statistical difference (*p* < 0.001) between the two groups.

## 4. Discussion

Dental implants are an effective and predictable treatment modality for the replacement of missing teeth in fully and partially edentulous patients. Nevertheless, despite high implant survival and success rates, failure might occur at an early or a late stage [18].

In our study, we focused on dental implants that were not osseointegrated before being loaded late with temporary or permanent prosthetic works. It is true that there are a multitude of causes that can negatively influence the healing of hard or soft tissue around dental implants.

In this study, which was carried out on a total of 213 patients in whom 810 dental implants were inserted over a period of 7 years, we sought to report the early lost implants in relation to the sex of the patients, the place of insertion of the dental implants, and the general conditions that counteract the lack of osseointegration of the dental implants, along with the vestibular bone additions and the diameter and length of the dental implants.

Implants lost early are most frequently associated with poor bone healing and the reduced primary stability of the implant [19,20,21,22]. Also, an increased temperature during the preparation of the implant site, which causes necrosis, as well as the wrong positioning of the implant, will cause the early loss of dental implants [22,23,24].

Certain systemic factors influence the body’s ability to heal, including the local inflammatory reaction. In this observational study, we obtained a non-osseointegration rate of dental implants of 2.71%, a percentage close to that obtained by Derks et al, van Steenberghe et al., Tolstunov, L, Quirynen, M et al., Chrcanovic, B.R., Roos-Jansaker AM et al., and Labriaga W et al. [6,25,26,27,28,29,30].

The risk of early loss of the implant, in the conducted study, was higher in women than in men, an aspect that contradicts the specialized literature, where a higher rate of early loss of the implant is related to men [31].

The number of inserted and non-osseointegrated implants in the maxilla and in the mandible was equal, in contradiction with a study conducted by Manzano G., which shows that the rate of early loss of implants is higher in the maxilla than in the mandible [32]. Although diabetes induces certain changes at the level of the organism, such as the reduction in the healing capacity, peripheral microvascular changes, and the decrease in the immune response, and at the level of the oral cavity, where it favors the appearance of carious lesions through the appearance of xerostomia and the installation of marginal periodontopathy, the current study did not highlight statistically significant correlations related to the early loss of dental implants and diabetic condition, confirming the findings in the specialized literature [33].

Cardiovascular diseases that are triggered by high levels of triglycerides can cause peri-implantitis-type conditions and, very rarely, the early loss of dental implants. In the study carried out here, only two dental implants were lost early, in accordance with the specialized literature [34].

Osteoclasts play an important role in the early and late stages of bone healing. Proton pump inhibitors are known to reduce osteoclast activity by increasing osteocalcin and osteoprotegerin levels [35,36]. It is also possible that proton pump inhibitors favor decreased bone turnover by preventing osteoclast V-ATPase, similar to how proton pump inhibitors inhibit gastric potassium hydrogen ATPase; however, higher levels [36], which have a negative effect on bone cells, [4,36] are most often associated with the early loss of dental implants [25,26,27].

In the current study, we did not find a direct link between antihypertensive medication with proton pump inhibitors and the early loss of dental implants. This is also confirmed by a recent study that does not indicate a direct causal relationship between medication with proton pump inhibitors and the early loss of dental implants [37].

The current study does not indicate that diabetes, cardiovascular diseases, and hypertension treated with proton pump inhibitors cause the early loss of dental implants, an aspect highlighted by the study by Alsaadi et al. [38].

No statistically significant relevance was found between early lost implants and vestibular bone augmentation, in opposition to the specialized literature which shows that there is a higher failure rate in the case of vestibular augmentations [39]. Toneti et al. have shown that an improvement in bone support can cause complications in the technique of inserting dental implants and that implants inserted in these areas do not have a long-term success rate like those placed in areas with natural bone [40].

The conducted study did not determine a statistically significant correlation between different narrow diameters (<3.5 mm) of dental implants and the standard one (3.5–4.5 mm), and large-diameter implants (>4.5 mm) were not inserted; similar results were obtained in the study conducted by Staedt H et al. [41]. So, the specialized literature claims that it is advisable to use implants with a larger diameter to reduce the transfer of occlusal stress to the surrounding alveolar bone [38].

Different studies have shown that implants with a short length (<8 mm) have a higher rate of early implant loss than implants longer than 10 mm [38,42,43].

## 5. Conclusions

The results obtained in the current study tend to be in agreement with the data from the specialized literature, confirming the fact that implant therapy is an effective oral rehabilitation method and can be used successfully, even in patients with associated different pathologies.

## Figures and Tables

**Table 1:** Number of dental implants by year.

Year	Number of Implants Placed
2017	33
2018	77
2019	108
2020	124
2021	159
2022	147
2023	162

**Table 2:** Number of inserted dental implants distributed by gender.

		Frequency	Percent	Valid Percent	Cumulative Percent
Valid	Male	389	48.0	48.0	48.0
	Female	421	52.0	52.0	100.0
	Total	810	100.0	100.0	

**Table 3:** The number of implants inserted according to the implant site.

		Frequency	Percent	Valid Percent	Cumulative Percent
Valid	Upper jaw right	194	24.0	24.0	24.0
	Upper jaw left	193	23.8	23.8	47.8
	Lower jaw right	236	29.1	29.1	76.9
	Lower jaw left	187	23.1	23.1	100.0
	Total	810	100.0	100.0	

**Table 4:** Distribution of dental implants by gender and implant site.

		Upper Jaw Right	Upper Jaw Left	Lower Jaw Right	Lower Jaw Left
Total implants number versus gender	Male	96	78	116	99
	Female	98	115	120	88
Total		194	193	236	187

**Table 5:** Distribution of implants with bone grafting in upper and lower jaw.

		Frequency	Percent	Valid Percent	Cumulative Percent
Valid	Upper-jaw vestibular bone grafting	103	12.7	12.7	12.7
	Upper-jaw vestibular no-bone grafting	365	45.1	45.1	57.8
	Lower-jaw vestibular bone grafting	63	7.8	7.8	65.6
	Lower-jaw vestibular no-bone grafting	279	34.4	34.4	100.0
	Total	810	100.0	100.0	

**Table 6:** Distribution of dental implants that benefited from vestibular bone addition to the jaw or mandible on the sexes.

		Frequency	Percent	Valid Percent	Cumulative Percent
Valid	Vestibular bone grafting, male upper jaw	45	5.6	5.6	5.6
	Vestibular bone grafting, female upper jaw	58	7.2	7.2	12.7
	No vestibular bone grafting, male upper jaw	136	16.8	16.8	29.5
	No vestibular bone grafting, female upper jaw	229	28.3	28.3	57.8
	Vestibular bone grafting, male lower jaw	23	2.8	2.8	60.6
	Vestibular bone grafting, female lower jaw	40	4.9	4.9	65.6
	No vestibular bone grafting, male lower jaw	105	13.0	13.0	78.5
	No vestibular bone grafting, female lower jaw	174	21.5	21.5	100.0

**Table 7:** Distribution of inserted implants by gender that were inserted at the maxillary sinus level with bone augmentation.

		Frequency	Percent	Valid Percent	Cumulative Percent
Valid	Male without external sinus lift	197	24.3	24.3	24.3
	Female without external sinus lift	426	52.6	52.6	76.9
	Male with external sinus lift	81	10.0	10.0	86.9
	Female with external sinus lift	106	13.1	13.1	100.0
	Total	810	100.0	100.0	

**Table 8:** Distribution of dental implants inserted by gender in the maxillary sinus to which bone addition was made on the left and right side.

		Frequency	Percent	Valid Percent	Cumulative Percent
Valid	Male without external sinus lift-Upper jaw right	135	16.7	16.7	16.7
	Female without external sinus lift-Upper jaw right	187	23.1	23.1	39.8
	Male with external sinus lift-Upper jaw right	39	4.8	4.8	44.6
	Female with external sinus lift-Upper jaw right	59	7.3	7.3	51.9
	Male without external sinus lift-Upper jaw left	122	15.1	15.1	66.9
	Female without external sinus lift-Upper jaw left	179	22.1	22.1	89.0
	Male with external sinus lift-Upper jaw left	42	5.2	5.2	94.2
	Female with external sinus lift-Upper jaw right	47	5.8	5.8	100.0
	Total	810	100.0	100.0	

**Table 9:** The dental implants that were inserted and were loaded with temporary dental work.

Immediately Loaded with Temporary Prosthetics	Upper Jaw		Lower Jaw	
	Male	Female	Male	Female
	9 (15.5%)	12 (20.7%)	12 (20.7%)	25 (43.10%)

**Table 10:** Distribution of implants that did not osseointegrate.

		Freaquance	Percent	Valid Percent	Cumulative Percent
	Upper jaw-male	3	13.6	13.6	13.6
Valid	Upper jaw-female	8	36.4	36.4	50.0
	Lower jaw-male	5	22.7	22.7	72.7
	Lower jaw-female	6	27.3	27.3	100.0
	Total	22	100.0	100.0	

**Table 11:** Distribution of non-osseointegrated implants by gender and type of bone augmentation performed.

		Freaquance	Percent	Valid Percent	Cumulative Percent
Valid	External sinus lift in male-upper jaw	3	13.6	13.6	13.6
	External sinus lift in female-upper jaw	4	18.2	18.2	31.8
	Vestibular bone grafting in male-upper jaw	1	4.5	4.5	36.4
	Vestibular bone grafting in female-upper jaw	2	9.1	9.1	45.5
	Vestibular bone grafting in male-lower jaw	3	13.6	13.6	59.1
	Vestibular bone grafting in female-lower jaw	4	18.2	18.2	77.3
	No bone grafting in male-upper jaw	1	4.5	4.5	81.8
	No bone grafting in female-upper jaw	1	4.5	4.5	86.4
	No bone grafting in male-lower jaw	2	9.1	9.1	95.5
	No bone grafting in female-lower jaw	1	4.5	4.5	100.0
	Total	22	100.0	100.0	

**Table 12:** Distribution of implants according to the connection with the prosthetic superstructure.

		Frequency	Percent	Valid Percent	Cumulative Percent
Valid	Male-platform switching implants-Upper jaw	87	10.7	10.7	10.7
	Female-platform switching implants-Upper jaw	99	12.2	12.2	23.0
	Male-non-platform switching implants-Upper jaw	89	11.0	11.0	34.0
	Female non-platform switching implants-Upper jaw	93	11.5	11.5	45.4
	Male-platform switching implants-Lower jaw	95	11.7	11.7	57.2
	Female-platform switching implants-Lower jaw	109	13.5	13.5	70.6
	Male-non-platform switching implants-Lower jaw	109	13.5	13.5	84.1
	Female non-platform switching implants-Lower jaw	129	15.9	15.9	100.0
	Total	810	100.0	100.0	

**Table 13:** Distribution of non-osseointegrated implants according to general conditions.

		Frequency	Percent	Valid Percent	Cumulative Percent
Valid	Diabetes in male-upper jaw	2	9.1	9.1	9.1
	Cardiovascular disease in male-upper jaw	1	4.5	4.5	13.6
	Arterial hypertension in male-upper jaw	1	4.5	4.5	18.2
	No general condition in male -upper jaw	2	9.1	9.1	27.3
	Diabetes in male- lower jaw	3	13.6	13.6	40.9
	Arterial hypertension in male-lower jaw	1	4.5	4.5	45.5
	No general condition in male -lower jaw	1	4.5	4.5	50.0
	Diabetes in female- lower jaw	5	22.7	22.7	72.7
	Diabetes in female- upper jaw	1	4.5	4.5	77.3
	Cardiovascular disease in female-upper jaw	1	4.5	4.5	81.8
	Arterial hypertension in female-lower jaw	1	4.5	4.5	86.4
	No general condition in female -lower jaw	2	9.1	9.1	95.5
	No general condition in female -upper jaw	1	4.5	4.5	100.0
	Total	22	100.0	100.0	

**Table 14:** Distribution of non-osseointegrated dental implants according to the diameter of the platform.

		Frequacy	Percent	Valid Percent	Cumulativ Percent
Valid	Narrow platform < 3.5 mm-male-upper jaw	3	13.6	13.6	13.6
	Narrow platform 3.5–4.5 mm-male-upper jaw	2	9.1	9.1	22.7
	Narrow platform < 3.5 mm female-upper jaw	2	9.1	9.1	31.8
	Narrow platform 3.5–4.5 mm-female-upper jaw	2	9.1	9.1	40.9
	Narrow platform < 3.5 mm-male-lower jaw	3	13.6	13.6	54.5
	Narrow platform 3.5–4.5 mm-male-lower jaw	4	18.2	18.2	72.7
	Narrow platform < 3.5 mm-female-lower jaw	4	18.2	18.2	90.9
	Narrow platform 3.5–4.5 mm-female-lower jaw	2	9.1	9.1	100.0
	Total	22	100.0	100.0	

**Table 15:** Distribution of non-osseointegrated dental implants according to the length of the implant.

		Frequency	Percent	Valid Percent	Cumulative Percent
Valid	8 mm	6	27.3	27.3	27.3
	10 mm	9	40.9	40.9	68.2
	11.5 mm	4	18.2	18.2	86.4
	13 mm	2	9.1	9.1	95.5
	16 mm	1	4.5	4.5	100.0
	Total	22	100.0	100.0	

## Data Availability

All data concerning this manuscript can be checked with the corresponding author at adina.cosarca@umfst.ro.
